# The Gut Microbiome–Endocannabinoidome Axis: A New Way of Controlling Metabolism, Inflammation, and Behavior

**DOI:** 10.1093/function/zqad003

**Published:** 2023-01-12

**Authors:** Cristoforo Silvestri, Vincenzo Di Marzo

**Affiliations:** Centre de Recherche de l’Institut de Pneumologie et Cardiologie de l’Université Laval, Département de médecine, Faculté de Médecine, Université Laval, Québec G1V 4G5, Canada; Institut sur la Nutrition et les Aliments Fonctionnels, Centre NUTRISS, Québec G1V 0A6, Canada; Centre de Recherche de l’Institut de Pneumologie et Cardiologie de l’Université Laval, Département de médecine, Faculté de Médecine, Université Laval, Québec G1V 4G5, Canada; Institut sur la Nutrition et les Aliments Fonctionnels, Centre NUTRISS, Québec G1V 0A6, Canada; Canada Research Excellence Chair on the Microbiome–Endocannabinoidome Axis in Metabolic Health (CERC-MEND), Université Laval, Québec G1V 0A6, Canada; École de nutrition, Faculté des sciences de l’agriculture et de l’alimentation (FSAA), Université Laval, Québec G1V 0A6, Canada

## The Endocannabinoidome and the Gut Microbiome: Not Two Worlds Apart

The endocannabinoidome (eCBome) is defined as an ensemble of (1) lipid mediators bearing chemical and, to some extent, biochemical and functional similarity with the two endogenous ligands of cannabinoid type-1 and type-2 (CB1 and CB2, respectively) receptors, that is, the endocannabinoids (eCBs) *N*-arachidonoyl-ethanolamine (anandamide, AEA) and 2-arachidonoyl-glycerol (2-AG); (2) the molecular targets for these mediators; and (3) their anabolic and catabolic enzymes.^1^ By definition, the eCBome thus includes the eCBs as well as several families of eCB-like molecules, and proteins controlling their levels or mediating their actions. Along with *N*-acyl-ethanolamines (NAEs) like anandamide, and 2-monoacyl-glycerols (2-MAGs) like 2-AG, other families include long-chain fatty acid primary amides; more than 200 hypothesized or actually identified *N*-acyl-amino acids; *N*-acylated neurotransmitters; and some bioactive oxidation products of the polyunsaturated members of each family.^1^ The mediators of the eCBome are usually produced “on demand,” ultimately from the remodeling and processing of membrane phospholipids, often following the elevation of intracellular calcium. They modulate the activity of more than 20 targets belonging to the G-protein-coupled receptor, ligand-activated ion channels, and peroxisome proliferator-activated receptor families, thereby regulating cell, tissue, and organismal functions as diverse as, for example, metabolism, inflammation, and behavior.^1^ Their relative composition in animal tissues is determined by several genetic, epigenetic, and environmental factors.

The gut microbiome (gμBiome), instead, is defined as the system of trillions of microorganisms (bacteria, archea, yeasts, and viruses, as well as, in some cases, unicellular prokaryotes) that populate the intestine of animals (the gut microbiota), together with their genes, proteins, and metabolites.^1^ The composition of the gμBiome, as in the case of the eCBome, is regulated by both innate and external factors, which are often the same that influence the eCBome, and in a way that two different individuals will never have the exact same taxonomic profile, especially at the genus or species level. Another strong analogy between these two complex systems consists in their similar implication in the regulation or, instead, when they are pathologically altered, dysregulation, of several physiological responses, including again energy metabolism, inflammation, and behavior.^1^ The gμBiome does so by producing, often following the processing of different nutrients, small molecule signals, such as short-chain fatty acids (SCFAs), various tryptophan metabolites, and secondary bile acids, among others, which can enter host circulation or affect its enteric nervous system.^1^ Recent evidence indicates that some commensal bacteria can also produce eCB-like molecules, similar to those of the eCBome, such as *N*-acylated ethanolamines, glycines, and amine neurotransmitters.^1^

Among the environmental factors strongly influencing the relative composition, and hence function, of both eCBome and gμBiome-derived signals, there is not only the caloric content but also, and perhaps more importantly, the fatty acid composition of the diet, and, particularly, the presence therein of omega-3 fatty acids. These usually lead to the prevalence of “beneficial” eCBome mediators and gμBiome taxa.^2^,^3^ While it is now clear that the effects of dietary fatty acids in either case can be direct as well as rapid and reversible,^2^,^3^ it is still not known to what extent such actions can also be the indirect consequence of effects of the the gμBiome on the eCBome and *vice versa*.

## The gμBiome and the eCBome Control Each Other

Indeed, ever increasing evidence exists in support of the possibility that the gμBiome controls eCBome signaling and function. This is shown, for example, by the fact that germ-free or antibiotic-treated mice present with different concentrations of eCBome receptors and/or mediators in both the gut and brain in a manner reversed or attenuated by fecal microbiota transfer (FMT) from conventionally raised mice.^4^,^5^ Additionally, probiotics were also suggested to produce some of their beneficial effects via eCBome signaling.^5^

Conversely, several studies have shown that eCBome mediators, such as NAEs^6^ and 2-MAGs^7^ can directly alter fecal microbiota composition *in vitro*, clearly via non-eCBome molecular targets present in bacteria, and *in vivo*, as, for example, in mice with genetically impaired inactivation of 2-MAGs,^7^ with potential functional consequences ranging from the control of intestinal inflammation^6^ to high fat diet (HFD)-induced obesity and dysmetabolism.^7^

Although these findings support the existence of a direct influence of the gμBiome over the eCBome and *vice versa*, further studies will be required to understand through what molecular mechanisms these reciprocal effects are exerted.

## The gμBiome–eCBome Axis in Peripheral Inflammation and Brain Function

A few pioneering studies have highlighted the functional importance of the gμBiome–eCBome axis in the physiological and pathological control not only of metabolism,^1^ as mentioned above, but also of peripheral inflammatory conditions and affective/motivational behaviors.

Fornelos and collaborators^6^ reported that NAE levels were higher in the feces of patients with inflammatory bowel disorders (IBDs) and in a mouse model of colitis. A cocktail of NAEs, including AEA, stimulated *in* vitro the growth of microbiota species that are usually more abundant, and inhibited that of species depleted, in IBDs. In particular, *Proteobacteria* bloomed and *Bacteroidetes* declined in the presence of NAEs. Kalkan et al.^8^ recently showed that in mdx mice, a model of Duchenne’s Muscular Dystrophy (DMD), the disease is associated with a significant alteration in gut microbiota composition, which results in the reduction of the plasma levels of SCFAs. Administration of one such molecule, sodium butyrate (NaB), rescued impaired muscle strength and autophagy, and prevented inflammation, all of which were due to excessive eCB signaling at CB1 receptors. NaB simultaneously reduced anandamide and CB1 receptor expression levels in mdx mouse skeletal muscle, and, in both murine and DMD human myoblasts, it exerted anti-inflammatory effects, promoted autophagy, and prevented excessive CB1 signaling by restoring normal levels of microRNAs that suppress CB1 expression.^8^

In antibiotic-induced despair in mice,^5^ the phosphorylation/sensitization of transient receptor potential vanilloid type-1 (TRPV1) channel, an eCBome receptor that participates in depression, was increased in the hippocampus, and the concentrations of the antidepressant eCBome mediators, *N*-arachidonoyl- and *N*-oleoyl-serotonin, which act as endogenous TRPV1 antagonists, were reduced in the gut. Treatment with *Lactobacillus* reduced gut dysbiosis, and reverted these eCBome signaling alterations, while significantly attenuating despair.^5^ Instead, in mice subjected to unpredictable chronic mild stress, despair behavior, and impaired neurogenesis could be transferred by FMT to unstressed recipient mice, which then exhibited these phenotypic alterations along with a decrease of brain 2-MAG and 2-AG levels, the latter possibly due to lower peripheral levels of the corresponding fatty acid precursors. The adverse effects of the transferred microbiota were counteracted by selectively enhancing central 2-AG levels with an inhibitor of 2-AG hydrolysis, or by supplementation with arachidonic acid or *Lactobacillus*, all of which also restored brain 2-AG levels and a gut microbiota composition similar to those of “unstressed” mice.^9^ Finally, again in mice, the microbiome-dependent production of NAEs and *N*-acyl-dopamines in the gut was found to stimulate the activity of TRPV1-expressing sensory neurons and to elevate dopamine levels in the ventral striatum during exercise. The essential role of this pathway in running motivation was shown by the finding that microbiome depletion as well as peripheral CB1 receptor antagonism, ablation of spinal afferent neurons, or dopamine blockade, all impaired exercise capacity.^10^

## Conclusions

**Figure 1.**
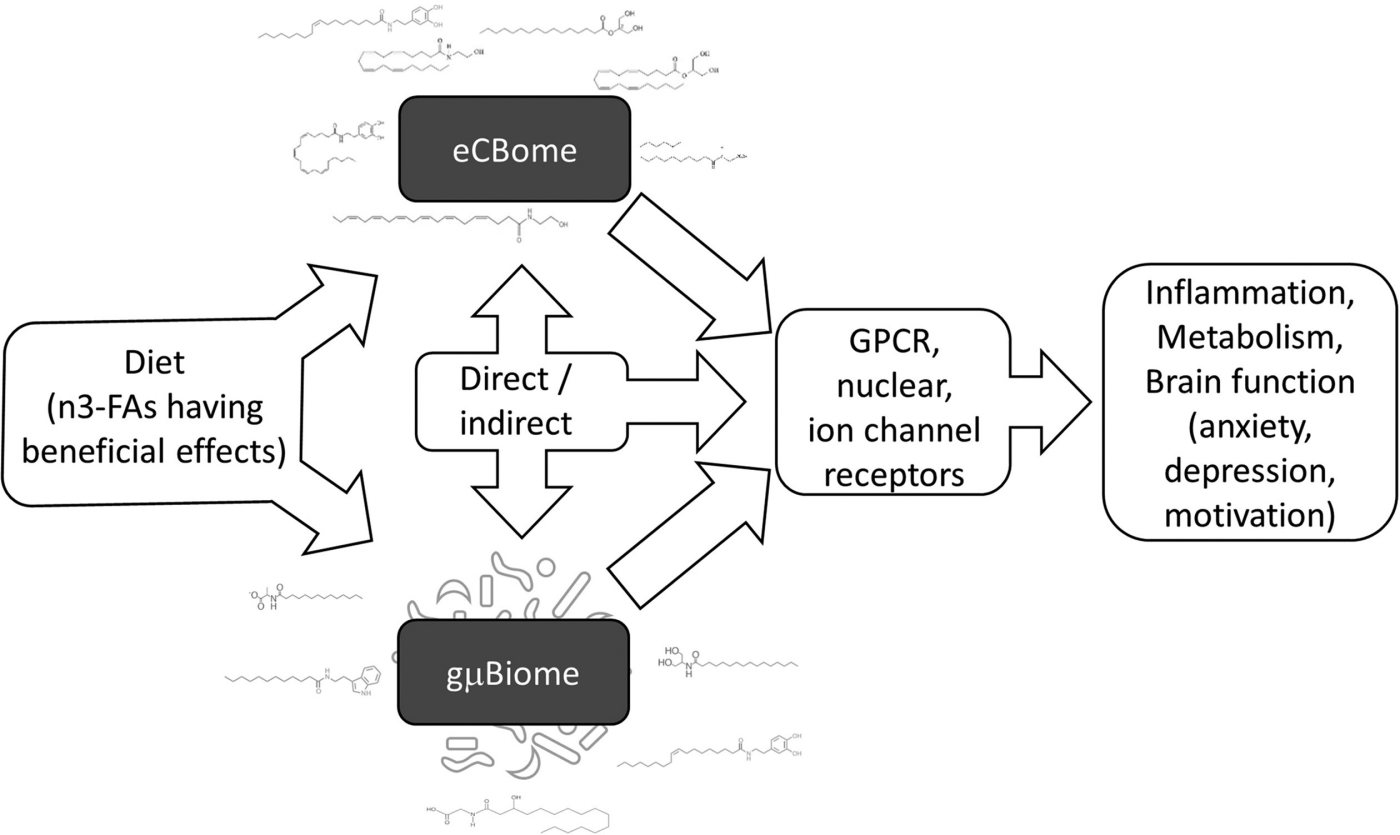
The eCBome and gμBiome are both responsive to similar environmental factors, including the diet, and especially its fatty acid composition, with omega-3 fatty acids generally reducing the levels of “detrimental” and increasing the levels of “beneficial” eCBome lipids as well as gμBiome bacterial taxa. Furthermore, it is increasingly evident that the eCBome and gμBiome, either by direct or indirect mechanisms, modify each other, including by producing similar bioactive lipids acting at overlapping receptors. Activity at these receptors modifies metabolic, immune, and brain function with effects on anxiety, depression, and motivation, among others.

In our opinion, there is to date enough evidence, coming from several laboratories and the use of multidisciplinary approaches, to support the participation of the gμBiome–eCBome axis in the control of energy metabolism, inflammation (including metabolic endotoxemia), and behaviour (Figure 1). However, most of the molecular mechanisms through which these two complex systems control each other are still unknown, and might include epigenetic as well as biochemical and pharmacological modulations.^6–10^ We believe that the gμBiome–eCBome axis will be more and more often the focus of studies aimed at understanding the role of small chemical signals in host–microbe communications of key functional importance.

## Funding

None declared.
